# Supplementary material for: MicroRNA Sequencing Analysis in Obstructive Sleep Apnea and Depression: Anti-Oxidant and MAOA-Inhibiting Effects of miR-15b-5p and miR-92b-3p through Targeting PTGS1-NF-κB-SP1 Signaling
Source: Antioxidants (Basel). 2021 Nov 22;10(11):1854. doi: 10.3390/antiox10111854 (PMC8614792; doi:10.3390/antiox10111854)
Supplement: Supplementary file 1 [file antioxidants-10-01854-s001.zip › antioxidants-1418755-supplementary.pdf]

microRNA sequencing analysis in obstructive sleep apnea and depression: anti-oxidant and MAOA-inhibiting effects of miR-15b-5p and miR-92b-3p through targeting PTGS1-NF-κB-SP1 signaling

Yung-Che Chen, Po-Yuan Hsu, Mao-Chang Su, Ting-Wen Chen, Chang-Chun Hsiao, Chien-Hung Chin, Chia-Wei Liou, Po-Wen Wang, Ting-Ya Wang, Yong-Yong Lin, Chiu Ping Lee, Meng-Chih Lin

Supplementary information

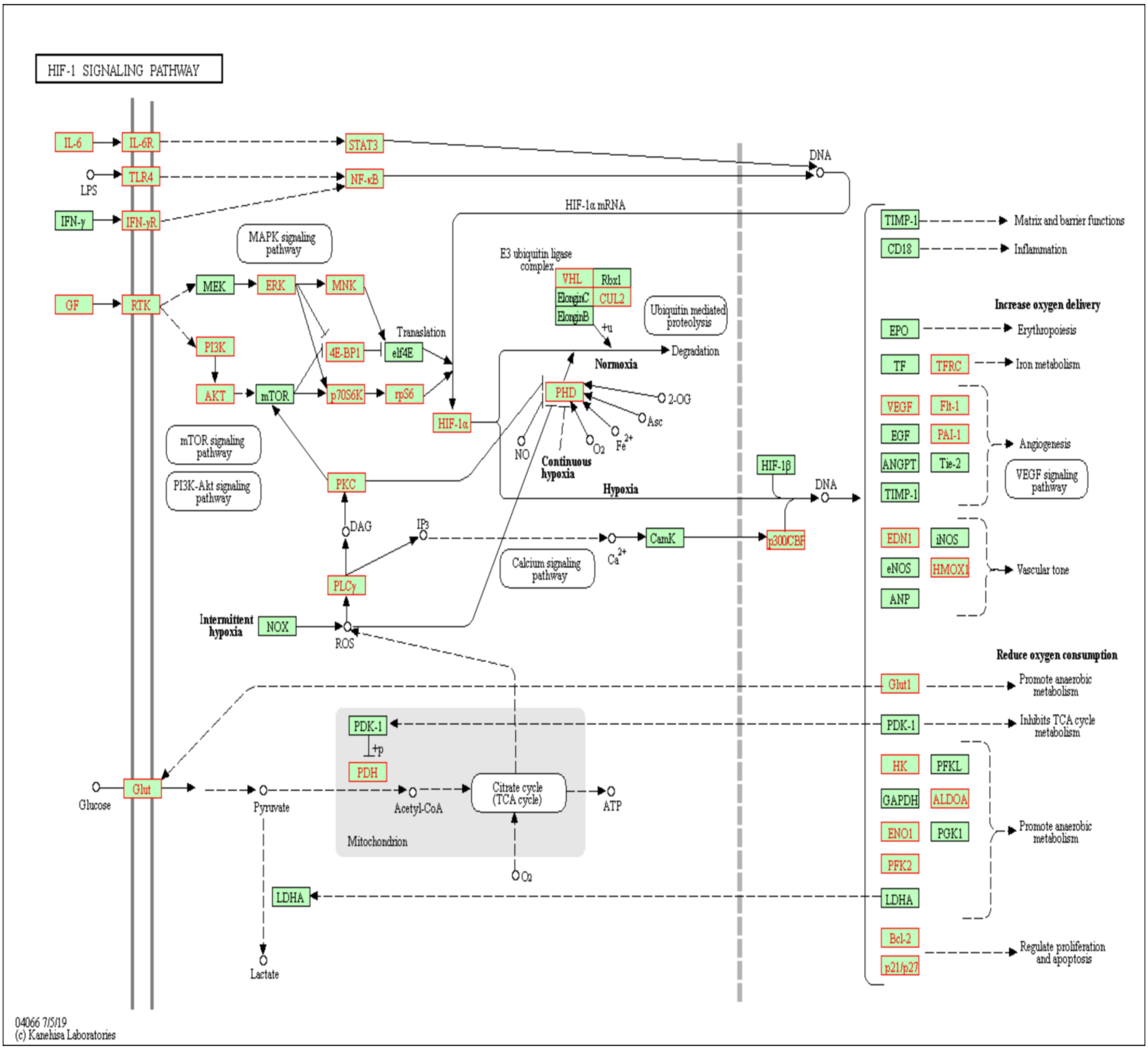

Supplementary Figure S1. The KEGG pathway of HIF-1 signaling is identified by over-representation analysis with the gene list derived from the validated target genes of the 22 OSA-related miRNAs (see Methods). The FDR-adjusted p-value of the pathways is 4.0E-4. Predicted target genes are shown in red letters and boxes with red lines.

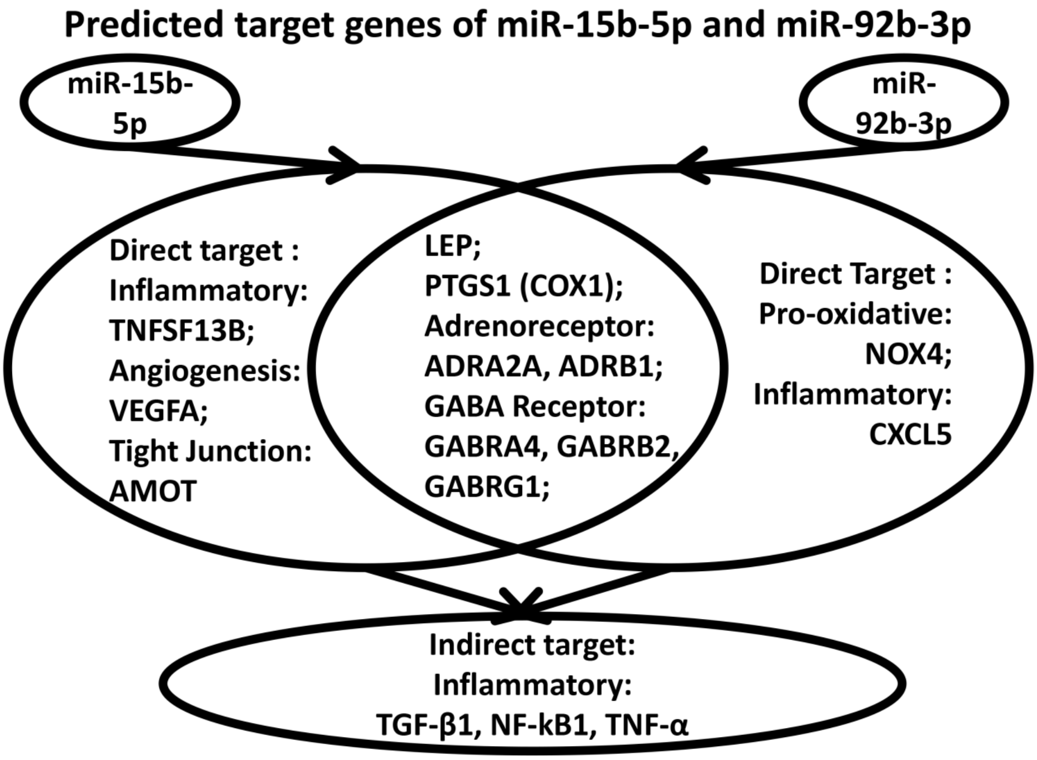

Supplementary Figure S2. Predicted direct and indirect target genes of miR-15b-5p and miR-92b-3p based on IPA and miRbase database.

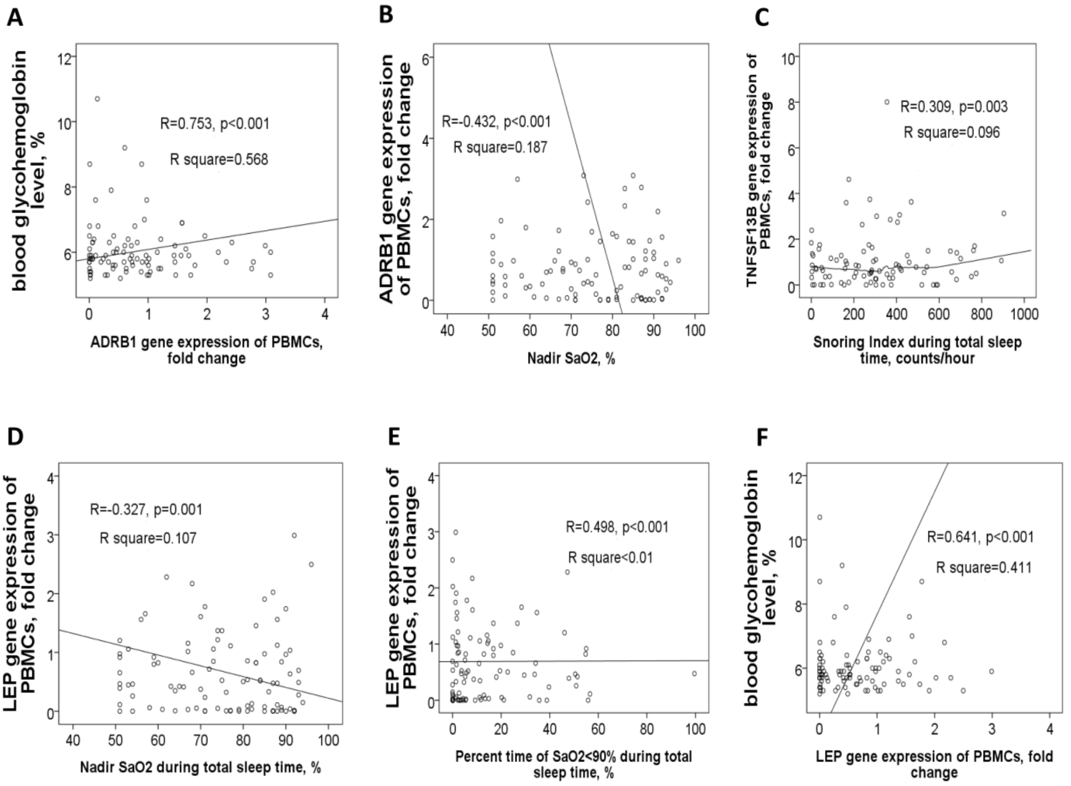

Supplementary Figure S3. Correlations of the predicted target gene expressions of miR-15b-5p/miR-92b-3p with sleep parameters and blood sugar control. Both (A) ADRB1 and (B) GABRB2 gene expressions were positively correlated with blood glycohemogobin (HbA1C) levels. (C) TNFSF13B gene expression was positively correlated with snoring index. LEP gene expression was negatively correlated with (D) nadir SaO2, and positively correlated with (E) percent time of SaO2<90% and (F) HbA1c.

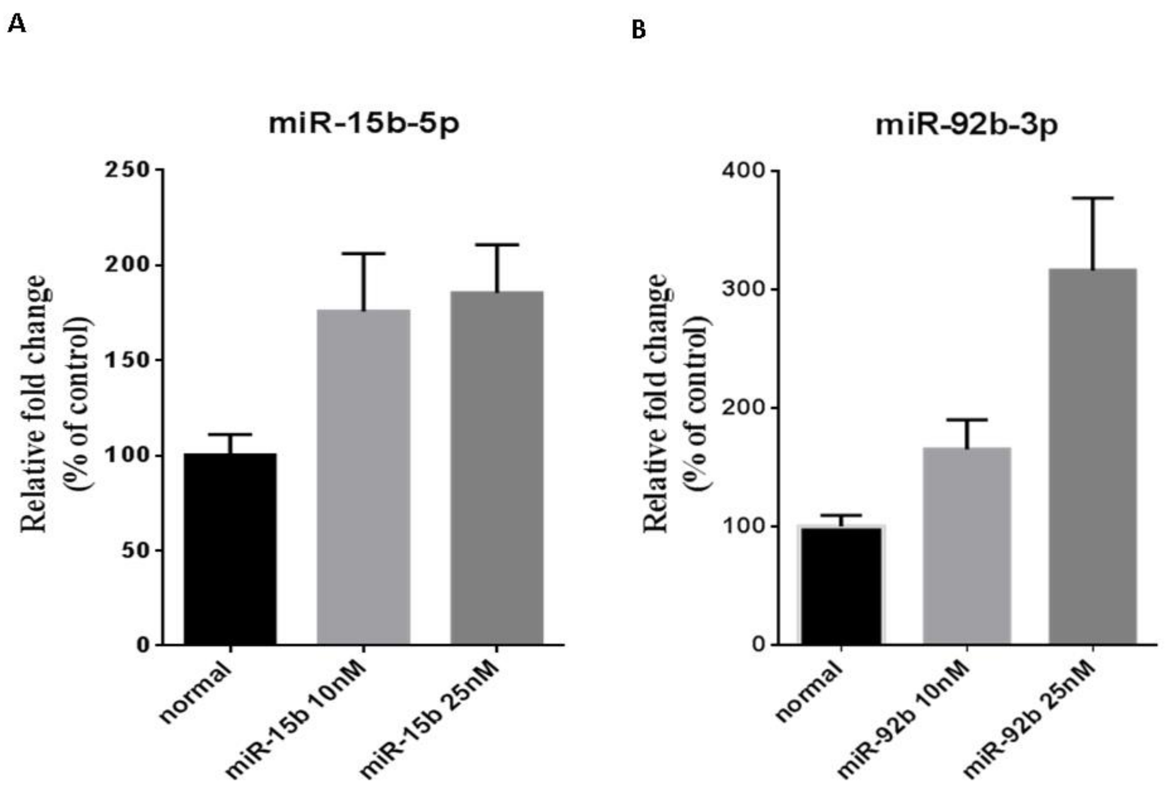

Supplementary Figure S4. Transfection efficiency of miR-15b-5p mimic and miR-92b-3p mimic in human umbilical vein endothelial cells (HUVEC). Transfection with either (A) miR-15b-5p mimic or (B) miR-92b-3p mimic in HUVEC at 10 nM or 25 nM resulted in 1.5 to 3 fold increases in their gene expression levels as compared with that of normal scrambler control.

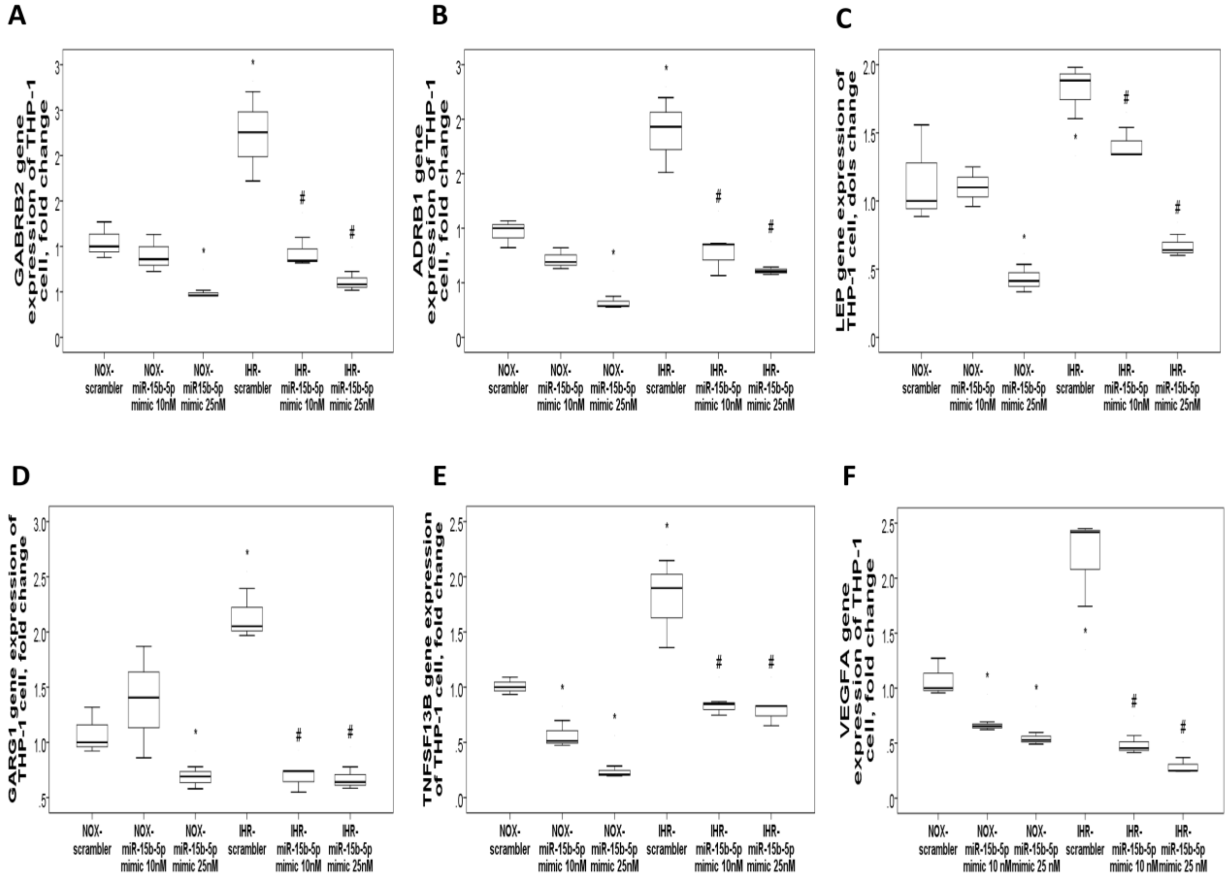

Supplementary Figure S5. MiR-15b-5p over-expression reversed intermittent hypoxia with re-oxygenation (IHR)-induced up-regulations of its target genes. Transfection with miR-15b-5p mimic at 25 nM in THP-1 cells reversed IHR-induced up-regulations of its predicted target genes, including (A) *GABRB2*, (B) *ADRB1*, (C) *LEP*, (D) *GARG1*, (E) *TNFSF13B*, and (F) *VEGFA*.

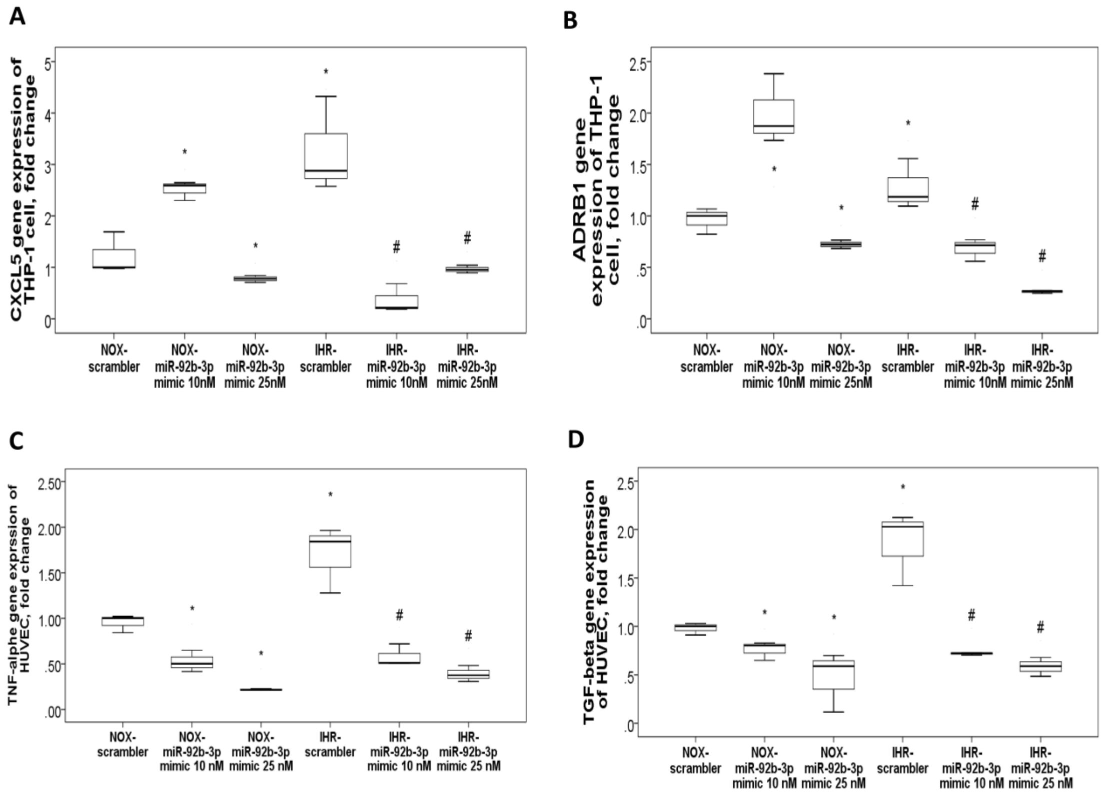

Supplementary Figure S6. MiR-92b-3p over-expression reversed intermittent hypoxia with re-oxygenation (IHR)-induced up-regulations of its target genes. Transfection with miR-92b-3p mimic at 25 nM in THP-1 cells reversed IHR-induced up-regulations of the (A) *CXCL5*, (B) *ADRB1*, (C) *TNF-α*, and (D) *TGF-β* genes.

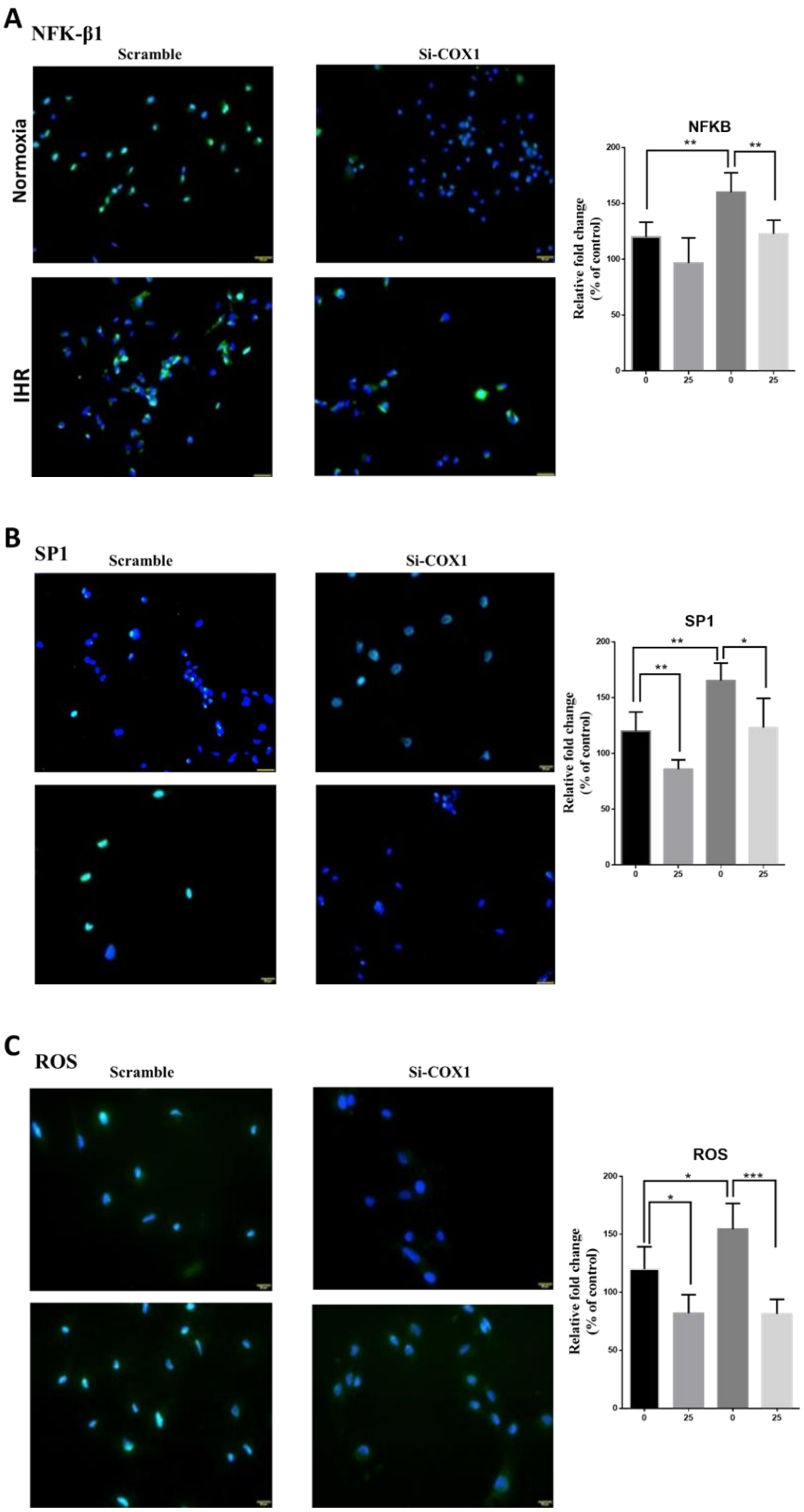

Supplementary Figure S7. PTGS1 (COX1) knock-down reversed intermittent hypoxia with re-oxygenation (IHR)-induced up-regulation of NF-κB/SP1, and oxidative stress. Representative micrographs of immunofluorescence staining in SH-SY5Y neuron cells with or without the knock-down of PTGS1 under IHR versus normoxic (NOX) condition are given for (A) NF-κB1, (B) SP1, and (C) reactive oxygen species (ROS). IHR resulted in over-expressions of NF-κB1, and SP1, and ROS over-production, all of which were re-versed with the knock-down of COX1. DAPI (blue) is used for staining the nuclei. Localizations of the three molecules are indicated in green. All the micrographs are a merge of the two staining. Quantified values are stratified based on the response to IHR stimuli and PTGS1 SiRNA transfection. Kruskal–Wallis test with post-hoc analysis was used for comparisons between four groups. *p<0.05. **p<0.01. ***p<0.001.

Supplementary Table S1. Probe sequences of the ten selected microRNAs and exogenous control *Caenorhabditis elegans* microRNA.

Probe ID	Gene name	Probe sequence
478293_mir	*cel-miR-39*	UCACCGGGUGUAAAUCAGCUUG
479802_mir	*hsa-miR-4433b-3p*	CAGGAGUGGGGGGUGGGACGU
478511_mir	*hsa-miR-133a-3p*	UUUGGUCCCCUUCAACCAGCUG
477984_mir	*hsa-miR-223-5p*	CGUGUAUUUGACAAGCUGAGUU
478313_mir	*hsa-miR-15b-5p*	UAGCAGCACAUCAUGGUUUACA
477823_mir	*hsa-miR-92b-3p*	UAUUGCACUCGUCCCGGCCUCC
477824_mir	*hsa-miR-148b-3p*	UCAGUGCAUCACAGAACUUUGU
477860_mir	*hsa-miR-16-5p*	UAGCAGCACGUAAAUAUUGGCG
478324_mir	*hsa-miR-335-5p*	UCAAGAGCAAUAACGAAAAAUGU
478575_mir	*hsa-let-7a-5p*	UGAGGUAGUAGGUUGUAUAGUU
477916_mir	*hsa-miR-145-5p*	GUCCAGUUUUCCCAGGAAUCCCU

Supplementary Table S2. Primer sequences of the predicted target mRNAs used for quantitative reverse-transcriptase polymerase chain reaction.

Gene Name		Sequence (5'-3')
TGF-beta	Forward	CAA GGG CTA CCA TGC CAA CT
	Reverse	AGG GCC AGG ACC TTG CTG
TNF-alpha	Forward	GAGGCCAAGCCCTGGTATG
	Reverse	CGGGCCGATTGATCTCAGC
BACE1	Forward	ACCAACCTTCGTTTGCCCAA
	Reverse	TCTCCTAGCCAGAAACCATCAG
JNK	Forward	TGTGTGGAATCAAGCACCTTC
	Reverse	AGGCGTCATCATAAAACTCGTTC
GSK3-beta	Forward	GGCAGCATGAAAGTTAGCAGA
	Reverse	GGCGACCAGTTCTCCTGAATC
INSR	Forward	CATCCGGGGATCACGACTG
	Reverse	ATCAGGTTGTAGAGGCCGAGT
NF-κB1	Forward	GAAGCACGAATGACAGAGGC
	Reverse	GCTTGGCGGATTAGCTCTTTT
ADRB1	Forward	ATCGAGACCCTGTGTGTCATT
	Reverse	GTAGAAGGAGACTACGGACGAG
ADRA2A	Forward	AGAAGTGGTACGTCATCTCGT
	Reverse	CGCTTGGCGATCTGGTAGA
CXCL5	Forward	AGCTGCGTTGCGTTTGTTTAC
	Reverse	TGGCGAACACTTGCAGATTAC
GABRG1	Forward	AAAAAGCCCTCCGTAGAAGTG
	Reverse	AGTTGAGTTCCGTAACCCTACA
GABRB2	Forward	GGGTGCCTGATACCTATTTCCT
	Reverse	GCGAATCATGCGGTTCTTAACA
GABRA4	Forward	ACAATGAGACTCACCATAAGTGC
	Reverse	AACTCCCGAATTTCAAAGGGC
PTGS1	Forward	CGCCAGTGAATCCCTGTTGTT
	Reverse	AAGGTGGCATTGACAAACTCC
PTGER4	Forward	CCGGCGGTGATGTTCATCTT
	Reverse	CCCACATACCAGCGTGTAGAA
TNFSF13B	Forward	GGGAGCAGTCACGCCTTAC
	Reverse	GATCGGACAGAGGGGCTTT
LEP	Forward	TGCCTTCCAGAAACGTGATCC
	Reverse	CTCTGTGGAGTAGCCTGAAGC
AMOT	Forward	CCTTAGAAGGAAGGCTGAGAAACT
	Reverse	GAGAAGAAGTAGGGCTGTGAGG
NOX4	Forward	CAG ATG TTG GGG CTA GGA TTG
	Reverse	GAG TGT TCG GCA CAT GGG TA
VEGFA	Forward	AGGGCAGAATCATCACGAAGT
	Reverse	AGGGTCTCGATTGGATGGCA

Supplementary Table S3. Enriched ingenuity pathway analysis for predicted target genes of the 22 differentially expressed miRNA.

**Ingenuity Canonical Pathways**	**-log(B-H p-value)***	**Ratio**	**Molecules: predicted target gene**
Senescence Pathway	19.6	0.254	ACVR1B,ACVR2A,ACVR2B,AKT3,ANAPC13,ASXL1,ASXL2,ATM,BMPR2,BRAF,CALM1 (includes others),CCNB1,CCND1,CDC23,CDC27,CDK6,CDKN1A,CDKN1B,CREBBP,CXCL8,DHCR24,E2F1,E2F3,E2F5,E2F7,EP300,HBP1,HIPK2,IL6,JUN,KRAS,MAP2K4,MAP3K7,MAPK1,MAPK14,MAPKAPK5,MDM2,NF1,NFAT5,NFATC3,NFKB1,NRAS,PARP1,PHF19,PIK3C2A,PIK3C2B,PIK3CA,PIK3R1,PPP2R2A,PPP2R5C,PTEN,RAP2A,RASSF5,RBL2,RPS6KA5,SERPINE1,SMAD2,SMAD3,SMAD4,SMAD5,SMAD7,SOD2,SQSTM1,TGFB1,TGFBR1,TGFBR2,TGFBR3,TP53,ZFP36L1
Molecular Mechanisms of Cancer	18	0.209	AKT3,APC,APH1A,ARHGEF10,ATM,BCL2,BCL2L11,BMP2,BMPR2,BRAF,CASP7,CBL,CCND1,CCND2,CDH1,CDK12,CDK19,CDK6,CDKN1A,CDKN1B,CREBBP,CRK,CTNNA1,CTNNB1,CTNND1,E2F1,E2F3,E2F5,E2F7,EP300,FAS,FOS,FOXO1,FZD6,GNA13,GNAI3,GSK3B,HIF1A,HIPK2,ITGA2,ITGA3,ITGA5,ITGA6,ITGAV,JUN,KRAS,MAP2K4,MAP3K7,MAPK1,MAPK14,MAPK8,MDM2,MYC,NF1,NFKB1,NRAS,PIK3C2A,PIK3C2B,PIK3CA,PIK3R1,PMAIP1,PRKAR2A,PRKCB,PRKDC,RAC1,RAP2A,RASA1,RBPJ,RHOB,SMAD2,SMAD3,SMAD4,SMAD5,SMAD7,TAB2,TCF3,TGFB1,TGFBR1,TGFBR2,TP53,WNT5A,XIAP
p53 Signaling	12.7	0.337	AKT3,ATM,BCL2,BIRC5,CCND1,CCND2,CCNG1,CDKN1A,CSNK1D,CTNNB1,E2F1,EP300,FAS,GSK3B,HIF1A,HIPK2,JMY,JUN,MAPK14,MAPK8,MDM2,MDM4,PIK3C2A,PIK3C2B,PIK3CA,PIK3R1,PMAIP1,PRKDC,PTEN,THBS1,TNFRSF10B,TP53,TP53INP1
Estrogen Receptor Signaling	11.4	0.19	AKT3,ATF2,BCL2,CARM1,CAV1,CCND1,CDKN1A,CFL2,CREB1,CREBBP,DDX5,EGFR,EIF2B2,EP300,FOS,FOXO1,GNA13,GNAI3,GNB1,GNG11,GSK3B,HIF1A,HSP90AA1,HSP90AB1,HSP90B1,IGF1R,JUN,KRAS,LIMK1,MAPK1,MED13,MED13L,MED6,MMP14,MYC,NCOA2,NCOA3,NCOR2,NFKB1,NR3C1,NRAS,PIK3C2A,PIK3C2B,PIK3CA,PIK3R1,PPP1R12A,PRKAA1,PRKAA2,PRKAB2,PRKAR2A,PRKCB,PRKDC,PTEN,RAP2A,RBFOX2,SOD2,SP1,TBL1XR1,TP53,TRRAP,VEGFA
Epithelial Adherens Junction Signaling	11.4	0.26	ACTB,ACTG1,ACTR2,ACTR3,ACVR1B,ACVR2A,ACVR2B,AFDN,AKT3,APC,ARPC5,BMPR2,CDH1,CLIP1,CRK,CTNNA1,CTNNB1,CTNND1,EGFR,FGFR1,KRAS,MET,MYH9,NOTCH2,NRAS,PARD3,PTEN,RAC1,RAP2A,SSX2IP,TCF3,TCF7L2,TGFBR1,TGFBR2,TGFBR3,TUBB,VCL,WASL,YES1
MSP-RON Signaling In Cancer Cells Pathway	11	0.271	AKT3,ATF2,BRAF,CCND1,CREB1,CREBBP,CTNNB1,EGFR,FLNA,FOS,GSK3B,HIF1A,ITGA6,JUN,KRAS,MAPK1,MET,MYC,NFKB1,NRAS,PIK3C2A,PIK3C2B,PIK3CA,PIK3R1,RAP2A,RPS6KA3,SMAD2,SP1,STAT3,TCF3,TCF7L2,VEGFA,YWHAB,YWHAE,YWHAQ,YWHAZ
Regulation Of The Epithelial Mesenchymal Transition By Growth Factors Pathway	10.7	0.229	AKT3,BRAF,CDH1,EGFR,FGF2,FGFR1,FOS,FOXO1,FRS2,GSK3B,HMGA2,ID2,IL6,JUN,KRAS,LATS1,LATS2,MAP2K4,MAP3K7,MAPK1,MAPK14,MAPK8,MET,NFKB1,NRAS,PARD6B,PIK3C2A,PIK3C2B,PIK3CA,PIK3R1,RAC1,RAP2A,SMAD2,SMAD3,SMAD4,STAT3,TCF3,TGFB1,TGFBR1,TGFBR2,TNFSF9,WWTR1,ZEB1
HGF Signaling	10.4	0.277	AKT3,ATF2,CCND1,CDKN1A,CRKL,FOS,IL6,ITGA2,ITGA3,ITGA5,ITGA6,ITGAV,JUN,KRAS,MAP2K4,MAP3K2,MAP3K7,MAP3K8,MAP3K9,MAPK1,MAPK8,MET,NRAS,PIK3C2A,PIK3C2B,PIK3CA,PIK3R1,PRKCB,PTGS2,PXN,RAC1,RAP2A,STAT3
HOTAIR Regulatory Pathway	10.3	0.244	AEBP2,AGO1,AGO2,AGO3,AGO4,AKT3,ATXN1,CDH1,CDKN1A,COL1A1,CREBBP,CTNNB1,EP300,H3-3A/H3-3B,IRF1,JARID2,KMT2A,KMT2C,MDM2,MET,MEX3B,MMP14,MYC,NFKB1,PIK3C2A,PIK3C2B,PIK3CA,PIK3R1,PTEN,RBBP7,REST,STAT3,STK38,TCF3,TCF7L2,TGFB1,WNT5A,XIAP
PTEN Signaling	10.2	0.257	AKT3,BCL2,BCL2L11,BMPR2,CBL,CCND1,CDKN1A,CDKN1B,CNKSR3,CSNK2A1,EGFR,FGFR1,FOXO1,GSK3B,IGF1R,ITGA2,ITGA3,ITGA5,

			ITGA6,ITGAV,KRAS,MAPK1,NFKB1,NRAS,NTRK2,OCRL,PIK3CA,PIK3R1,PTEN,RAC1,RAP2A,SYNJ1,TGFBR1,TGFBR2,TGFBR3
TGF-β Signaling	10.2	0.302	ACVR1B,ACVR2A,ACVR2B,BCL2,BMP2,BMPR2,CREBBP,EP300,FOS,JUN,KRAS,MAP2K4,MAP3K7,MAPK1,MAPK14,MAPK8,NRAS,PMEPA1,RAP2A,SERPINE1,SKI,SMAD2,SMAD3,SMAD4,SMAD5,SMAD7,TGFB1,TGFBR1,TGFBR2
ILK Signaling	9.86	0.222	ACTB,ACTG1,AKT3,ATF2,BMP2,CCND1,CDH1,CFL2,CREB1,CREBBP,CTNNB1,FLNA,FOS,GSK3B,HIF1A,IRS2,IRS4,ITGB8,JUN,MAP2K4,MAPK1,MAPK8,MYC,MYH9,NFKB1,PIK3C2A,PIK3C2B,PIK3CA,PIK3R1,PPP1R12A,PPP2R2A,PPP2R5C,PTEN,PTGS2,PXN,RAC1,RHOB,RICTOR,RPS6KA5,VCL,VEGFA
ERK/MAPK Signaling	9.86	0.214	ATF2,BRAF,CREB1,CREBBP,CRK,CRKL,DUSP1,DUSP2,DUSP4,DUSP6,FOS,H3-3A/H3-3B,ITGA2,ITGA3,ITGA5,ITGA6,ITGAV,KRAS,MAPK1,MAPKAPK5,MKNK2,MYC,NRAS,PIK3C2A,PIK3C2B,PIK3CA,PIK3R1,PPP1CC,PPP1R12A,PPP2R2A,PPP2R5C,PRKAR2A,PRKCB,PXN,RAC1,RAP2A,RPS6KA5,STAT1,STAT3,TLN1,YWHAB,YWHAQ,YWHAZ
PPARα/RXRα Activation	9.77	0.223	ACOX1,ACVR1B,ACVR2A,ACVR2B,BMPR2,CAND1,CKAP5,CREBBP,EP300,FASN,GPD2,HSP90AA1,HSP90AB1,HSP90B1,IL6,JUN,KRAS,MAP2K4,MAP3K7,MAPK1,MAPK14,MAPK8,NCOA3,NCOR2,NFKB1,NR2C2,NRAS,PRKAA1,PRKAA2,PRKAB2,PRKAR2A,PRKCB,RAP2A,SMAD2,SMAD3,SMAD4,TGFB1,TGFBR1,TGFBR2,TGFBR3
Sumoylation Pathway	9.71	0.287	ARHGDIA,CBX4,CDH1,CREBBP,EP300,FAS,FOS,JUN,MAP2K4,MAPK8,MDM2,MYB,NFKB1,NR3C1,RAC1,RAN,RANBP2,RANGAP1,RHOB,SENP1,SENP5,SERBP1,SMAD4,SP1,SP3,TP53,XIAP,ZEB1,ZNF217
FAT10 Cancer Signaling Pathway	9.3	0.413	ACKR3,ACVR1B,ACVR2A,ACVR2B,AKT3,BMPR2,CTNNB1,GSK3B,IL6,NFKB1,SMAD2,SMAD3,SMAD4,STAT3,TGFB1,TGFBR1,TGFBR2,TGFBR3,TP53
Regulation of the Epithelial-Mesenchymal Transition Pathway	9.02	0.209	AKT3,APC,APH1A,BRAF,CDH1,CTNNB1,EGFR,FGF2,FGFR1,FRS2,FZD6,GSK3B,HIF1A,HMGA2,ID2,KRAS,MAP2K4,MAPK1,MET,NFKB1,NOTCH2,NRAS,PARD6B,PIK3C2A,PIK3C2B,PIK3CA,PIK3R1,RAP2A,RBPJ,SMAD2,SMAD3,SMAD4,STAT3,TCF3,TCF7L2,TGFB1,TGFBR1,TGFBR2,WNT5A,ZEB1
HIF1α Signaling	8.91	0.204	AKT3,BRAF,CCNG2,CDKN1A,CREBBP,EGLN1,EP300,FGF2,HIF1A,HIF1AN,HK1,HSP90AA1,HSPA1A/HSPA1B,HSPA5,HSPA8,IL6,JUN,KRAS,MAP2K4,MAPK1,MDM2,MET,MKNK2,MMP14,NRAS,PIK3C2A,PIK3C2B,PIK3CA,PIK3R1,PKM,PRKCB,RACK1,RAN,RAP2A,SAT1,SERPINE1,SLC2A3,STAT3,TGFB1,TP53,VEGFA
Coronavirus Pathogenesis Pathway	8.91	0.233	BCL2,BCL2L11,CCND1,CXCL8,E2F1,E2F3,E2F5,E2F7,EP300,FOS,IL6,JUN,KPNB1,MAP2K4,MAPK1,MAPK14,MAPK8,MAVS,NFKB1,NPM1,PTGS2,RBL2,RPS14,SERPINE1,SMAD3,SMAD4,STAT1,STAT3,TGFB1,TGFBR1,TGFBR2,TNPO1,TNPO2,TP53
IGF-1 Signaling	8.82	0.269	AKT3,CCN2,CSNK2A1,FOS,FOXO1,IGF1R,IRS2,JUN,KRAS,MAPK1,MAPK8,NRAS,PIK3C2A,PIK3C2B,PIK3CA,PIK3R1,PRKAR2A,PXN,RAP2A,RASA1,SOCS5,SOCS6,SOCS7,STAT3,YWHAB,YWHAE,YWHAQ,YWHAZ
Hypoxia Signaling in the Cardiovascular System	8.79	0.319	ATF2,ATM,BIRC6,CREB1,CREBBP,CSNK1D,EP300,HIF1A,HIF1AN,HSP90AA1,HSP90AB1,HSP90B1,JUN,MDM2,PTEN,TP53,UBE2D3,UBE2G1,UBE2Q2,UBE2V2,UBE2W,UBE2Z,VEGFA
Actin Cytoskeleton Signaling	8.67	0.194	ABI2,ACTB,ACTG1,ACTR2,ACTR3,APC,ARHGAP35,ARPC5,CFL2,CRK,CRKL,EZR,FGF2,FLNA,GNA13,GNG12,ITGA2,ITGA3,ITGA5,ITGA6,ITGAV,KRAS,LIMK1,MAPK1,MSN,MYH9,MYLK3,NCKAP1,NRAS,PIK3C2A,PIK3C2B,PIK3CA,PIK3R1,PPP1R12A,PXN,RAC1,RAP2A,SLC9A1,TIAM1,TLN1,TRIO,VCL,WASL
PDGF Signaling	8.65	0.291	ABL2,CAV1,CRK,CRKL,CSNK2A1,EIF2AK2,FOS,JUN,KRAS,MAP2K4,MAPK1,MAPK8,MYC,NRAS,OCRL,PIK3C2A,PIK3C2B,PIK3CA,PIK3R1,PRKCB,RAP2A,RASA1,STAT1,STAT3,SYNJ1

PI3K/AKT Signaling	8.54	0.208	AKT3,BCL2,CCND1,CDKN1A,CDKN1B,CTNNB1,FOXO1,GSK3B,HSP90AA1,HSP90AB1,HSP90B1,IL6ST,ITGA2,ITGA3,ITGA5,ITGA6,ITGAV,KRAS,MAP3K8,MAPK1,MCL1,MDM2,NFKB1,NRAS,OCRL,PIK3CA,PIK3R1,PPP2R2A,PPP2R5C,PTEN,PTGS2,RAP2A,SYNJ1,TP53,YWHAB,YWHAE,YWHAQ,YWHAZ
Aryl Hydrocarbon Receptor Signaling	8.49	0.234	AHR,ATM,CCNA2,CCND1,CCND2,CDK6,CDKN1A,CDKN1B,E2F1,EP300,FAS,FOS,HSP90AA1,HSP90AB1,HSP90B1,IL6,JUN,MAPK1,MAPK8,MDM2,MYC,NCOA2,NCOA3,NCOR2,NFIA,NFIB,NFIC,NFKB1,RBL2,SP1,TGFB1,TP53
T Cell Exhaustion Signaling Pathway	8.41	0.213	ACVR1B,ACVR2A,ACVR2B,AKT3,BMPR2,CD274,FOS,FOXO1,FOXP1,IL6,IRF4,JUN,KRAS,MAP2K4,MAPK1,MAPK8,NFAT5,NFATC3,NRAS,PIK3C2A,PIK3C2B,PIK3CA,PIK3R1,PPP2R2A,PPP2R5C,PRDM1,RAP2A,SMAD2,SMAD3,STAT1,STAT3,TGFB1,TGFBR1,TGFBR2,TGFBR3,VEGFA
Wnt/β-catenin Signaling	8.36	0.212	ACVR1B,ACVR2A,ACVR2B,AKT3,APC,BMPR2,BTRC,CCND1,CDH1,CREBBP,CSNK1A1,CSNK1D,CSNK1E,CSNK2A1,CTNNB1,EP300,FZD6,GSK3B,JUN,MAP3K7,MDM2,MYC,PPP2R2A,PPP2R5C,SOX11,SOX4,SOX5,TCF3,TCF7L2,TGFB1,TGFBR1,TGFBR2,TGFBR3,TLE4,TP53,WNT5A
Protein Kinase A Signaling	8.31	0.156	AKAP10,AKAP11,AKAP9,ANAPC13,ATF2,BRAF,CALM1 (includes others),CDC23,CDC27,CREB1,CREBBP,CTNNB1,DUSP1,DUSP2,DUSP3,DUSP4,DUSP5,DUSP6,FLNA,GDE1,GNA13,GNAI3,GNB1,GNG11,GNG12,GSK3B,H3-3A/H3-3B,ITPR1,MAPK1,MYLK3,NFAT5,NFATC3,NFKB1,PALM2AKAP2,PDE3A,PDE4D,PDE7A,PPP1CC,PPP1R12A,PRKAR2A,PRKCB,PTEN,PTGS2,PTP4A1,PTPN14,PTPN4,PTPRD,PTPRJ,PXN,SMAD3,SMAD4,TCF3,TCF7L2,TGFB1,TGFBR1,TGFBR2,YWHAB,YWHAE,YWHAQ,YWHAZ
Integrin Signaling	8.23	0.195	ACTB,ACTG1,ACTR2,ACTR3,AKT3,ARF3,ARF4,ARHGAP5,ARPC5,ASAP1,BRAF,CAV1,CRK,CRKL,GSK3B,ITGA2,ITGA3,ITGA5,ITGA6,ITGAV,ITGB8,KRAS,MAP2K4,MAPK1,MAPK8,MYLK3,NRAS,PIK3C2A,PIK3C2B,PIK3CA,PIK3R1,PPP1R12A,PTEN,PXN,RAC1,RAP2A,RHOB,TLN1,VCL,WASL
HIPPO signaling	8.21	0.286	BTRC,CSNK1D,CSNK1E,CUL1,LATS1,LATS2,MOB1A,NF2,PARD3,PPP1CC,PPP1R12A,PPP2R2A,PPP2R5C,SMAD2,SMAD3,SMAD4,SMAD5,STK4,TEAD1,WWTR1,YWHAB,YWHAE,YWHAQ,YWHAZ
Rac Signaling	8.1	0.242	ABI2,ACTR2,ACTR3,ARPC5,CDK5R1,CFL2,ELK4,ITGA2,ITGA3,ITGA5,ITGA6,ITGAV,JUN,KRAS,LIMK1,MAP2K4,MAPK1,MAPK8,NCKAP1,NFKB1,NRAS,PARD3,PIK3C2A,PIK3C2B,PIK3CA,PIK3R1,RAC1,RAP2A,TIAM1
Cell Cycle: G1/S Checkpoint Regulation	8.1	0.318	ATM,BTRC,CCND1,CCND2,CDK6,CDKN1A,CDKN1B,CUL1,E2F1,E2F3,E2F5,E2F7,FOXO1,GSK3B,MDM2,MYC,RBL2,SMAD3,SMAD4,TGFB1,TP53
Reelin Signaling in Neurons	8.03	0.24	ACTR2,ACTR3,AFDN,AKT3,APP,ARHGEF10,ARPC5,CDK5R1,CRK,CRKL,FRK,GSK3B,ITGA3,ITGA5,LIMK1,MAP1B,MAP2K4,MAP3K9,MAPK1,MAPK8,PAFAH1B1,PAFAH1B2,PIK3C2A,PIK3C2B,PIK3CA,PIK3R1,RAC1,WASL,YES1
RAN Signaling	8.02	0.647	CSE1L,KPNA1,KPNA2,KPNA5,KPNA6,KPNB1,RAN,RANBP2,RANGAP1,TNPO1,XPO1
Role of Tissue Factor in Cancer	7.93	0.243	AKT3,CCN2,CFL2,CXCL8,EGFR,F3,FRK,GNA13,ITGA3,ITGA6,ITGAV,KRAS,LIMK1,MAPK1,MAPK14,NRAS,PIK3C2A,PIK3C2B,PIK3CA,PIK3R1,PTEN,RAC1,RAP2A,RPS6KA3,RPS6KA5,TP53,VEGFA,YES1
Cardiac Hypertrophy Signaling (Enhanced)	7.93	0.142	ACVR1B,ACVR2A,ACVR2B,AKT3,ATF2,ATP2A2,BMPR2,CALM1 (includes others),CTNNB1,CXCL8,EIF2B2,EP300,FGF2,FGFR1,FZD6,GDE1,GNA13,GNAI3,GNB1,GNG11,GSK3B,IGF1R,IL6,IL6ST,ITGA2,ITGA3,ITGA5,ITGA6,ITGAV,ITPR1,JUN,KRAS,MAP2K4,MAP3K2,MAP3K20,MAP3K7,MAP3K8,MAP3K9,MAPK1,MAPK14,MAPK8,MEF2D,MKNK2,MYC,NFAT5,NFATC3,NFKB1,NRAS,PDE3A,PDE4D,PDE7A,PIK3C2A,PIK3C2B,PIK3CA,PIK3R1,PRKAR2A,PRKCB,PTEN,PTGS2,RAP2A,RCAN1,RPS6KA5,STAT3,TGFB1,TGFBR1,TGFBR2,TGFBR3,TNFSF9,WNT5A
Glucocorticoid Receptor Signaling	7.55	0.143	ACTB,AKT3,ARID1A,BCL2,BCL2L11,CAV1,CDKN1A,CDKN1C,CREB1,CREBBP,CREBZF,CXCL8,DUSP1,EGFR,EP300,FOS,GTF2H1,HSP90A

			A1,HSP90AB1,HSP90B1,HSPA1A/HSPA1B,HSPA5,HSPA8,IL6,IL6ST,JUN,KRAS,MAP2K4,MAP3K7,MAPK1,MAPK14,MAPK8,MYC,NCOA2,NCOA3,NCOR2,NFAT5,NFATC3,NFKB1,NR3C1,NRAS,PHF10,PIK3C2A,PIK3C2B,PIK3CA,PIK3R1,POLR2D,PRKAA1,PRKAA2,PRKAB2,PTGS2,RAC1,RAP2A,RPS6KA5,SERPINE1,SMAD2,SMAD3,SMAD4,SMARCD1,STAT1,STAT3,TGFB1,TGFBR1,TGFBR2,TSC22D3
STAT3 Pathway	7.54	0.222	BCL2,BMPR2,CDKN1A,EGFR,FGF2,FGFR1,IGF1R,IL6ST,KRAS,MAP2K4,MAP3K20,MAP3K21,MAP3K9,MAPK1,MAPK14,MAPK8,MYC,NRAS,NTRK2,RAC1,RAP2A,SOCS5,SOCS6,SOCS7,STAT3,TGFB1,TGFBR1,TGFBR2,TGFBR3,VEGFA
Tumor Microenvironment Pathway	7.54	0.2	AKT3,BCL2,BRAF,CCND1,CD274,COL1A1,CXCL8,FAS,FGF2,FOS,FOXO1,HIF1A,IL6,ITGA5,JUN,KRAS,MAPK1,MMP14,MYC,NFKB1,NRAS,PIK3C2A,PIK3C2B,PIK3CA,PIK3R1,PTGS2,RAC1,RAP2A,SLC16A1,SLC1A4,SLC2A3,STAT3,TGFB1,TIAM1,VEGFA
ERK5 Signaling	7.41	0.292	ATF2,CREB1,CREBBP,EGFR,ELK4,FOS,GNA13,IL6ST,KRAS,MAP3K2,MAP3K8,MEF2D,MYC,NRAS,RAP2A,RPS6KA3,RPS6KA5,YWHAB,YWHAE,YWHAQ,YWHAZ
Signaling by Rho Family GTPases	7.24	0.172	ACTB,ACTG1,ACTR2,ACTR3,ARHGEF10,ARPC5,CDH1,CFL2,CLIP1,EZR,FOS,GNA13,GNAI3,GNB1,GNG11,GNG12,ITGA2,ITGA3,ITGA5,ITGA6,ITGAV,JUN,LIMK1,MAP2K4,MAP3K20,MAP3K21,MAP3K9,MAPK1,MAPK8,MSN,NFKB1,PARD3,PIK3C2A,PIK3C2B,PIK3CA,PIK3R1,PPP1R12A,RAC1,RHOB,SEPTIN11,SEPTIN2,SLC9A1,WASL
Cell Cycle: G2/M DNA Damage Checkpoint Regulation	7.21	0.347	ATM,BTRC,CCNB1,CDKN1A,CUL1,EP300,HIPK2,MDM2,MDM4,PPM1D,PRKDC,TP53,WEE1,YWHAB,YWHAE,YWHAQ,YWHAZ
Synaptogenesis Signaling Pathway	7.19	0.16	ACTR2,ACTR3,AFDN,AKT3,AP2B1,ARPC5,ATF2,BRAF,CALM1 (includes others),CDH1,CREB1,CREBBP,CRK,CRKL,CTNNB1,CTNND1,DNAJC5,EFNA1,EIF4EBP2,EPHA4,GOSR1,GSK3B,HSPA8,ITPR1,KRAS,LIMK1,MAP1B,MAPK1,MAPK14,MARCKS,NAP1L1,NRAS,NTRK2,PAFAH1B1,PIK3C2A,PIK3C2B,PIK3CA,PIK3R1,PRKAR2A,RAB5B,RAB5C,RAC1,RAP2A,STX16,THBS1,TIAM1,TLN1,WASL,YES1
Prolactin Signaling	7.17	0.272	CREBBP,EP300,FOS,IRF1,JUN,KRAS,MAPK1,MYC,NR3C1,NRAS,PIK3C2A,PIK3C2B,PIK3CA,PIK3R1,PRKCB,RAP2A,SOCS5,SOCS6,SOCS7,SP1,STAT1,STAT3
Sertoli Cell-Sertoli Cell Junction Signaling	7.16	0.189	ACTB,ACTG1,AFDN,AKT3,ATF2,CDH1,CLDN1,CTNNA1,CTNNB1,GSK3B,ITGA2,ITGA3,ITGA5,ITGA6,ITGAV,JUN,KRAS,MAP2K4,MAP3K2,MAP3K20,MAP3K7,MAP3K8,MAP3K9,MAPK1,MAPK14,MAPK8,NRAS,PRKAR2A,PTEN,RAC1,RAP2A,SPTBN1,TGFBR3,TJP1,TUBB,VCL
RAR Activation	7.05	0.188	ACTB,AKT3,ARID1A,BMP2,CARM1,CREBBP,CSNK2A1,DUSP1,EP300,FOS,GTF2H1,JUN,MAP2K4,MAPK1,MAPK14,MAPK8,NCOR2,NFKB1,NSD1,PARP1,PHF10,PIK3CA,PIK3R1,PRKAR2A,PRKCB,PTEN,RAC1,REL,SMAD2,SMAD3,SMAD4,SMAD5,SMAD7,SMARCD1,TGFB1,VEGFA

*-log(B-H p-value) >= 1.3 indicates a significant pathway.

Supplementary Table S4. Enriched ingenuity pathway analysis pathways for the intersection of the predicted target genes of miR-15b-5p and miR-92b-3p.

Ingenuity Canonical Pathways	-log(B-H p-value)*	Ratio	Molecules
Molecular Mechanisms of Cancer	5	0.0636	CDK6,CHEK1,CREBBP,CRK,CTNNB1,E2F3,FADD,FZD6,GNAI3,GNAQ,GRB2,GSK3B,ITGA6,MAP2K4,MAP3K7,MAPK1,MDM2,PRKAR2A,RBPJ,SMAD2,SMAD3,SMAD7,SYNGAP1,TAB2,TCF3
Regulation of the Epithelial-Mesenchymal Transition Pathway	3.2	0.0733	CTNNB1,FGF2,FZD6,GRB2,GSK3B,HMGA2,MAP2K4,MAPK1,PARD6B,RBPJ,SMAD2,SMAD3,SMURF1,TCF3
TGF-β Signaling	3.2	0.104	CREBBP,GRB2,MAP2K4,MAP3K7,MAPK1,SKI,SMAD2,SMAD3,SMAD7,SMURF1
Regulation Of The Epithelial Mesenchymal Transition By Growth Factors Pathway	3.2	0.0745	FGF2,GRB2,GSK3B,HMGA2,LATS1,LATS2,MAP2K4,MAP3K7,MAPK1,PARD6B,SMAD2,SMAD3,SMURF1,TCF3

EIF2 Signaling	2.82	0.066	AGO4,EIF2B2,EIF3A,EIF4G2,EIF5B,GRB2,GSK3B,HNRNPA1,MAPK1,PPP1CB,PTBP1,RPLP0,RPS14,RPS3A
Senescence Pathway	2.82	0.0588	ANAPC13,ASXL2,CDC27,CDK6,CHEK1,CREBBP,E2F3,MAP2K4,MAP3K7,MAPK1,MDM2,PHF19,RBL2,SMAD2,SMAD3,SMAD7
Protein Kinase A Signaling	2.63	0.0494	AKAP10,ANAPC13,CDC27,CREBBP,CTNNB1,DUSP6,GNAI3,GNAQ,GNB2,GSK3B,ITPR1,MAPK1,PPP1CB,PRKAR2A,PTPRJ,SMAD3,TCF3,YWHAB,YWHAE
Inhibition of ARE-Mediated mRNA Degradation Pathway	2.63	0.082	AGO4,CNOT4,DDX6,MAP3K7,MAPK1,PABPN1,PRKAR2A,XRN1,YWHAB,YWHAE
Coronavirus Pathogenesis Pathway	2.63	0.0753	E2F3,MAP2K4,MAPK1,NPM1,RBL2,RPS14,RPS3A,SMAD3,TNPO1,TNPO2,TRAF3
Telomere Extension by Telomerase	2.51	0.267	HNRNPA1,HNRNPA2B1,TNKS2,XRCC5
BMP signaling pathway	2.51	0.0964	CREBBP,GRB2,MAP2K4,MAP3K7,MAPK1,PRKAR2A,SMAD7,SMURF1
MSP-RON Signaling In Cancer Cells Pathway	2.51	0.0752	CREBBP,CTNNB1,GRB2,GSK3B,ITGA6,MAPK1,SMAD2,TCF3,YWHAB,YWHAE
Insulin Receptor Signaling	2.46	0.0735	CRK,CRKL,EIF2B2,GRB2,GSK3B,IRS4,MAPK1,PPP1CB,PRKAR2A,SYNJ1
Role of PKR in Interferon Induction and Antiviral Response	2.36	0.0783	FADD,HSPA1A/HSPA1B,IRF1,MAP2K4,MAP3K7,MAPK1,NPM1,TAB2,TRAF3
IL-1 Signaling	2.34	0.087	GNAI3,GNAQ,GNB2,MAP2K4,MAP3K7,MAPK1,PRKAR2A,TAB2
PPARα/RXRα Activation	2.18	0.0615	CAND1,CREBBP,FASN,GNAQ,GRB2,MAP2K4,MAP3K7,MAPK1,PRKAR2A,SMAD2,SMAD3
PI3K/AKT Signaling	2.14	0.0601	CTNNB1,GRB2,GSK3B,IL6ST,ITGA6,MAPK1,MCL1,MDM2,SYNJ1,YWHAB,YWHAE
HOTAIR Regulatory Pathway	2.14	0.0641	AGO4,CREBBP,CTNNB1,IRF1,JARID2,KMT2A,KMT2C,MDM2,STK38,TCF3
Ephrin Receptor Signaling	2.13	0.0588	ADAM10,CREBBP,CRK,CRKL,GNAI3,GNAQ,GNB2,GRB2,ITGA6,MAPK1,SDC2
HIPPO signaling	2.01	0.0833	LATS1,LATS2,PPP1CB,SMAD2,SMAD3,YWHAB,YWHAE
FGF Signaling	2.01	0.0833	CREBBP,CRK,CRKL,FGF2,GRB2,ITPR1,MAPK1
Huntington's Disease Signaling	1.94	0.0513	CDK5R1,CLTC,CREBBP,GNAQ,GNB2,GRB2,HSPA1A/HSPA1B,HTT,ITPR1,MAP2K4,MAPK1,NSF
ERK/MAPK Signaling	1.94	0.0547	CREBBP,CRK,CRKL,DUSP6,GRB2,ITGA6,MAPK1,PLA2G12A,PPP1CB,PRKAR2A,YWHAB
Cell Cycle: G1/S Checkpoint Regulation	1.9	0.0909	CDK6,E2F3,GSK3B,MDM2,RBL2,SMAD3
Role of NFAT in Cardiac Hypertrophy	1.85	0.0521	GNAI3,GNAQ,GNB2,GRB2,GSK3B,IL6ST,ITPR1,MAP2K4,MAP3K7,MAPK1,PRKAR2A
PFKFB4 Signaling Pathway	1.85	0.109	CREBBP,FGF2,MAP2K4,MAPK1,PRKAR2A
Reelin Signaling in Neurons	1.84	0.0661	CDK5R1,CRK,CRKL,GSK3B,MAP1B,MAP2K4,MAPK1,PAFAH1B1
Melatonin Signaling	1.82	0.0857	GNAI3,GNAQ,MAP2K4,MAPK1,PRKAR2A,RORA
ERK5 Signaling	1.78	0.0833	CREBBP,ELK4,GNAQ,IL6ST,YWHAB,YWHAE
Cell Cycle: G2/M DNA Damage Checkpoint Regulation	1.78	0.102	CHEK1,MDM2,WEE1,YWHAB,YWHAE
Myc Mediated Apoptosis Signaling	1.75	0.1	CREBBP,FADD,MCL1,MDM2,PRKAR2A
Sumoylation Pathway	1.71	0.0693	CBX4,CREBBP,MAP2K4,MDM2,RAN,SP3,ZNF217
Human Embryonic Stem Cell Pluripotency	1.69	0.0606	CTNNB1,FGF2,FZD6,GSK3B,SMAD2,SMAD3,SMAD7,TCF3
Regulation of eIF4 and p70S6K Signaling	1.69	0.0556	AGO4,EIF2B2,EIF3A,EIF4G2,GRB2,ITGA6,MAPK1,RPS14,RPS3A
Hepatic Fibrosis Signaling Pathway	1.69	0.0407	CREBBP,CTNNB1,FGF2,FZD6,GNAI3,GSK3B,ITGA6,MAP2K4,MAP3K7,MAPK1,PRKAR2A,SMAD2,SMAD3,SMAD7,TCF3
Paxillin Signaling	1.64	0.066	ARF1,CRK,GRB2,ITGA6,MAP2K4,MAPK1,VCL
Cardiac Hypertrophy Signaling	1.62	0.0468	CREBBP,EIF2B2,GNAI3,GNAQ,GNB2,GRB2,GSK3B,MAP2K4,MAP3K7,MAPK1,PRKAR2A
GNRH Signaling	1.62	0.0529	CREBBP,GNAI3,GNAQ,GRB2,ITPR1,MAP2K4,MAP3K7,MAPK1,PRKAR2A

RAN Signaling	1.62	0.176	KPNA5,RAN,TNPO1
Wnt/β-catenin Signaling	1.62	0.0529	APPL1,CREBBP,CTNNB1,FZD6,GNAQ,GSK3B,MAP3K7,MDM2,TCF3
Synaptogenesis Signaling Pathway	1.62	0.0423	CREBBP,CRK,CRKL,CTNNB1,GRB2,GSK3B,ITPR1,MAP1B,MAPK1,NSF,PAFAH1B1,PRKAR2A,SYNGAP1
Integrin Signaling	1.61	0.0488	ARF1,CRK,CRKL,GRB2,GSK3B,ITGA6,MAP2K4,MAPK1,PPP1CB,VCL
Insulin Secretion Signaling Pathway	1.61	0.046	AGO4,CREBBP,EIF2B2,EIF4G2,GNAQ,ITPR1,MAPK1,NSF,PRKAR2A,SPCS3,TCF3
Opioid Signaling Pathway	1.55	0.0451	CLTC,CREBBP,CTNNB1,GNAI3,GSK3B,ITPR1,MAP2K4,MAPK1,OGFR,PRKAR2A,SLC12A5
Factors Promoting Cardiogenesis in Vertebrates	1.55	0.0552	CREBBP,CTNNB1,FZD6,GSK3B,MAP2K4,MAP3K7,SMAD2,TCF3
PDGF Signaling	1.55	0.0698	CRK,CRKL,GRB2,MAP2K4,MAPK1,SYNJ1
Endocannabinoid Developing Neuron Pathway	1.55	0.0609	CREBBP,CTNNB1,GNAI3,GSK3B,MAP2K4,MAPK1,PRKAR2A
Necroptosis Signaling Pathway	1.43	0.0523	FADD,FKBP1A,MAP3K7,MDM2,PLA2G12A,RBL2,TAB2,VDAC2
Actin Cytoskeleton Signaling	1.42	0.045	ABI2,CRK,CRKL,FGF2,GRB2,ITGA6,MAPK1,PPP1CB,SLC9A1,VCL
α-Adrenergic Signaling	1.4	0.0638	GNAI3,GNAQ,GNB2,ITPR1,MAPK1,PRKAR2A
Sertoli Cell-Sertoli Cell Junction Signaling	1.4	0.0474	CTNNB1,GSK3B,ITGA6,MAP2K4,MAP3K7,MAPK1,PRKAR2A,SYMPK,VCL
ATM Signaling	1.38	0.0625	CBX5,CHEK1,CREBBP,MAP2K4,MDM2,RNF168

*-log(B-H p-value) >= 1.3 indicates a significant pathway.
